# Metal Nanoparticles in Laser Bioprinting

**DOI:** 10.3390/nano11102584

**Published:** 2021-09-30

**Authors:** Vyacheslav Zhigarkov, Ivan Volchkov, Vladimir Yusupov, Boris Chichkov

**Affiliations:** 1 Federal Scientific Research Centre “Crystallography and Photonics”, Russian Academy of Sciences, Pionerskaya St., 2, 108840 Moscow, Russia; vzhigarkov@gmail.com (V.Z.); volch2862@gmail.com (I.V.); chichkov@iqo.uni-hannover.de (B.C.); 2 Institute of Quantum Optics, Leibniz University of Hanover, Welfengarten 1, D-30167 Hanover, Germany

**Keywords:** laser bioprinting, Ti nanoparticles, gel microdroplets

## Abstract

Laser bioprinting is a promising method for applications in biotechnology, tissue engineering, and regenerative medicine. It is based on a microdroplet transfer from a donor slide induced by laser pulse heating of a thin metal absorption film covered with a layer of hydrogel containing living cells (bioink). Due to the presence of the metal absorption layer, some debris in the form of metal nanoparticles is printed together with bioink microdroplets. In this article, experimental investigations of the amount of metal nanoparticles formed during the laser bioprinting process and transported in bioink microdroplets are performed. As metal absorption layers, Ti films with the thickness in the range of 25–400 nm, produced by magnetron spattering, were applied. Dependences of the volume of bioink microdroplets and the amount of Ti nanoparticles within them on the laser pulse fluence were obtained. It has been experimentally found that practically all nanoparticles remain in the hydrogel layer on the donor slide during bioprinting, with only a small fraction of them transferred within the microdroplet (0.5% to 2.5%). These results are very important for applications of laser bioprinting since the transferred metal nanoparticles can potentially affect living systems. The good news is that the amount of such nanoparticles is very low to produce any negative effect on the printed cells.

## 1. Introduction

The method of laser-induced forward transfer (LIFT) of a microscopic amount of matter as a result of laser-pulsed heating is widely used in various fields of science and technology [1]. LIFT-based bioprinting is used in biofabrication to create artificial biological tissues [2,3,4,5,6] and in laser engineering of microbiological systems (LEMS) to isolate difficult-to-cultivate microorganisms [7,8,9,10].

In laser bioprinting and LEMS technologies, a hydrogel layer with living cells or microorganisms (bioink) is covered on a transparent donor glass with a metal absorbing layer [11,12,13]. Pulsed laser heating of the absorbing layer leads to the formation of a rapidly expanding bubble in the hydrogel layer and then a thin jet of bioink, from which a microdroplet with living cells or microorganisms is then separated and transferred to an acceptor slide [14].

In laser bioprinting, cells or microorganisms are exposed to several physical factors: direct laser radiation; sharp jumps in temperature and pressure at the surface of the laser absorbing film; and high dynamic loads associated with the initial acceleration and the subsequent “landing” of the microdroplet on the acceptor slide [9,13,15,16,17]. One of such factors that can affect laser printed cells or microorganisms is a possible transfer of nanoparticles formed during laser heating and ablation of metal films [18]. Information on the presence, concentration, and size of nanoparticles in the laser-printed bioink microdroplets is of great practical importance. This article is focused on experimental investigations of nanoparticles formed during laser bioprinting with a special emphasis on LEMS technologies.

## 2. Materials and Methods

**Figure 1:**
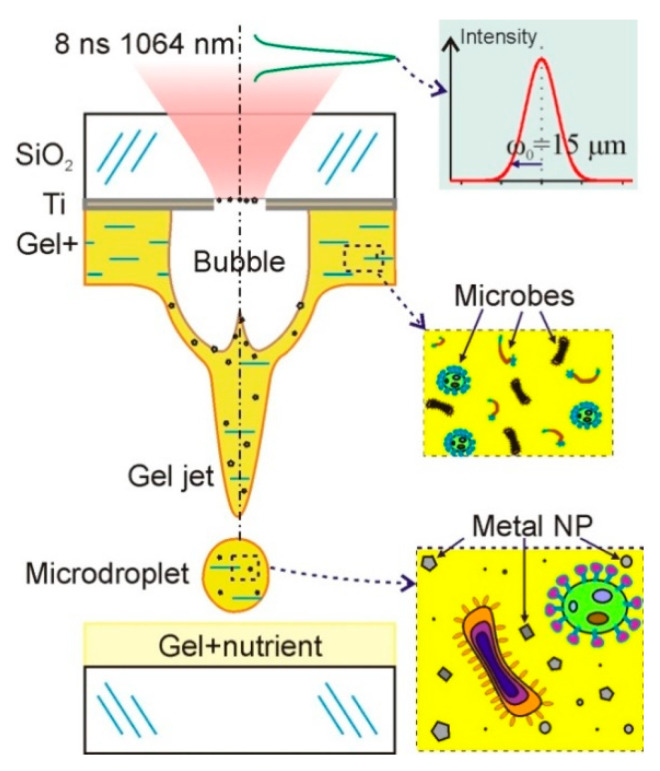
Schematic diagram of bioprinting setup. A donor slide is shown with a titanium layer on which a relatively thick layer of hydrogel with living microorganisms is deposited. Under the action of a short laser pulse, a gel microdroplet is transferred to the acceptor slide with a nutrient. This microdroplet contains both microorganisms and nanoparticles of the destroyed metal film.

Diagrams of a standard laser bioprinting setup are presented in many articles (see, for example, [9,13,14]). The basic setup elements used in our work on LEMS are a pulsed laser, a system for laser beam forming, and a scanner with a lens focusing laser beam on a donor slide. Figure 1 schematically shows a donor slide with an absorbing titanium film and a gel layer of 200 μm thickness with microorganisms. Laser pulse absorption in a titanium film ultimately leads to a transfer of a gel microdroplet with microorganisms to an acceptor slide. Due to the destruction of the metal film, the droplet also contains Ti nanoparticles.

In our LEMS experiments, a bioprinting system with a YLPM-1-4 × 200-20-20 pulsed fiber laser (NTO “IRE-Polyus”, Russia) providing Gaussian pulses at a wavelength λ of 1064 nm, pulse duration *τ* of 8 ns, and M2 < 1.5 is used. Laser radiation is focused onto the absorbing layer using a LscanH-10-1064 Galvano scanning head (AtekoTM, Moscow, Russia) with an F-theta SL-1064-110-160 lens (Ronar-Smith, Singapore) with a focal length of 160 mm. This system forms a laser spot with a diameter of 30 μm (the radius of the laser waist ω_0_ is 15 μm), positioning it with an accuracy of several microns in the horizontal XY plane. Laser pulse energies used in our experiments are in the range of *E* = 1 to 44 μJ. This corresponds to the peak laser fluence of *F_0_ = 2E/(πω_0_^2^) =* 0.28–12.4 J/cm^2^ and the average fluence of *F =* 0.14–6.2 J/cm^2^. The operating range of energies allowing stable transfer of single droplets is between 15–30 μJ [17]. An S310C meter (Thorlabs, Newton, NJ, USA) was used to control the energy of the pulsed laser radiation. The results are presented as the mean and standard deviation.

Most of the experiments were conducted with the donor glass plates (Menzel, Thermo Fisher Scientific) on which Ti films with a thickness of 50 nm were deposited using magnetron sputtering. The thickness of the deposited film was controlled using atomic force microscopy methods. Ti films are very often used as laser absorbing materials in laser bioprinting. Titanium is frequently applied in laser-induced forward transfer (LIFT) due to its unique industrial advantages (low density, high specific strength, and high corrosion resistance) [19]. We used donor slides with titanium film because of the much stronger adhesion of Ti to glass compared to gold films. The second reason is that our previous experiments [18] showed that Ti nanoparticles (TiNP) transferred with microorganisms during laser bioprinting have a significantly lower negative effect compared to Au nanoparticles.

In this paper, two series of experiments are performed. First, parameters of hydrogel microdroplets transferred from the donor slide on the surface of the acceptor slide, amount and size distribution of the nanoparticles within these microdroplets, as well as sizes of laser-ablated holes in the absorbing Ti film at different laser pulse energies are studied. A hydrogel layer with a thickness of 200 ± 20 μm was deposited on the absorbing Ti film using a blade coater. The hydrogel was obtained by dissolving hyaluronic acid sodium salt (HySilk, Contipro) in deionized water with a mass concentration of 2% [14]. The volume of gel microdroplets was determined using an optical image of microdroplets on the acceptor slide, taking into account the measured contact angle [14].

In the second series of experiments, results of the action of laser pulses on the donor slide and the acceptor sapphire plate, placed in close contact with the surface of Ti film are investigated. Special attention is paid to the metal nanoparticles left on the donor slide and transferred to the acceptor plate, and their sizes.

After laser exposure, the samples are examined by SEM, AFM, and optical microscopy. We use a PHENOM ProX (Phenom-World, The Netherlands) and an HRM-300 Series optical 3D microscopes (Huvitz, Korea). Analysis of the surface morphology of the studied samples is performed by scanning electron microscopy (SEM) using a JEOL JCM-6000 PLUS SEM in the secondary electron mode (15 kV), equipped with an X-ray energy dispersive spectrometer. The surfaces of the Ti film and sapphire plate are studied by X-ray phase analysis with a MiniFlex 600 X-ray diffractometer (Rigaku, Japan) using Cu-Kα radiation. The surface relief in the region of laser action is investigated by atomic force microscopy using the Solver Pro M complex (ZAO Nanotechnology MDT, Russia) with the contact method. All results are presented as averages and standard deviations. When constructing trends, the method of least squares is applied.

## 3. Results and Discussion

**Figure 2:**
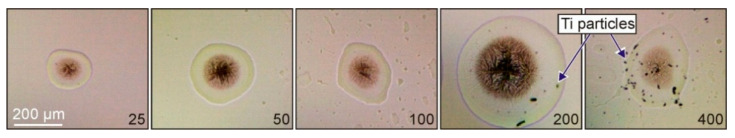
Optical micrographs of dried gel microdroplets on the acceptor slide produced with the same laser irradiation parameters (*τ* = 8 ns, *E* = 20 μJ, and *F* = 2.8 J/cm^2^) and different thicknesses of the Ti film on the donor slide. The numbers show the thickness of the Ti film in nanometers. In the last two images, Ti particles both inside and outside microdroplets can be seen.

In the first series of experiments, we studied the parameters of the laser-printed hydrogel microdroplets on the surface of the acceptor slide and investigated nanoparticles inside these droplets. Typical optical micrographs of dried gel microdroplets on the acceptor slide are shown in Figure 2. The size of this microdroplets is determined by the energy of laser pulses, the thickness of Ti film, and the characteristics of the hydrogel layer [14]. They were obtained with different thicknesses of Ti film and a gel layer with 2% solution of hyaluronic acid and thickness of 200 ± 20 μm. At the center of the dried droplets, there is a dark rounded area associated with the crystallization of hyaluronic acid.

Figure 2 shows that the size of the formed microdroplet significantly depends on the thickness of the absorbing Ti film. The microdroplet diameter gradually increases with the increasing thickness of the absorbing film, reaches a maximum, and then decreases. The initial increase in the microdroplet size is associated with the gradual increase in the fraction of the absorbed laser energy by the Ti film. The observed decrease in the microdroplet size for 400 nm thick Ti film is associated with the decreasing temperature which can be reached in this film.

From the microdroplet images, one can conclude that Ti film thicknesses of 25 and 50 nm are better for the generation of small and neat microdroplets. At the film thickness of 100 nm, splashing of hydrogel can be observed. For 200 and 400 nm Ti films, the transferred particles are clearly visible on the donor slide.

**Figure 3:**
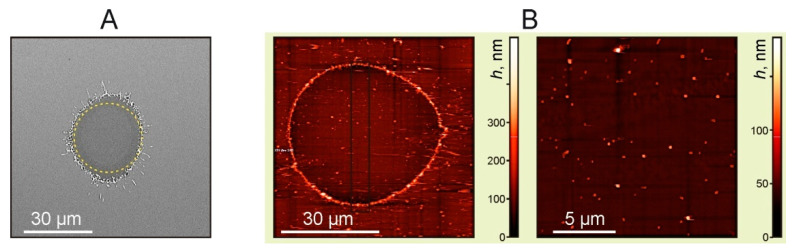
Images of the Ti film with a thickness of 50 nm after laser exposure. (**A**) SEM image. The dotted line shows a circle with a diameter of 30 μm, *E* = 7 μJ (*F* = 1 J/cm^2^). (**B**) AFM images of the ablated areas, *E* = 24 μJ (*F* = 3.4 J/cm^2^).

The results of the Ti film (with a 50 nm thickness) destruction on the donor slide, due to laser ablation, can be clearly seen in Figure 3. One can see a hole produced in the Ti film and a visible absence of nanoparticles inside this hole. The hole edges are melted, as can be seen in the SEM image. A more detailed AFM image (Figure 3B) shows that nanoparticles can be observed and are present inside the hole. Most of these nanoparticles in the horizontal plane have a size of 190 ± 36 nm and in the vertical plane their size is determined by the film thickness of ~50 nm.

The available SEM images of holes in Ti films can be used for estimates of the maximum temperature obtained at the hole center. In the absence of phase transitions and heat conduction losses, the resulted Ti film temperature is directly proportional to the laser pulse fluence [20,21]. In this case, the temperature profile is determined by the Gaussian laser intensity distribution in the focal spot area with the radius ω_0_. Since the edges of the hole in the Ti film were melted (Figure 3A), at the end of the laser pulse, the temperature reached in this area should be of the order of the titanium melting temperature, *T_mTi_* = 1670 °C. In the inner hole region, part of the laser energy was spent on melting and evaporating of the Ti film. Taking this into account, we obtain the following temperature distribution (neglecting the room temperature):

(1) $$T_{mTi}=(\Delta T_{0}+q_{1}/c_{1}+q_{2}/c_{2})\cdot \exp(-2{r_{0}}^{2}/{\omega_{0}}^{2}),$$

where Δ*T*_0_ is the temperature jump at the hole center, *q*_1_ is the specific heat of fusion, *q*_2_ is the specific heat of vaporization, *c*_1_ is the heat capacity of titanium at the melting point (*T_mTi_* = 1670 °C), *c*_2_ is the heat capacity of titanium at the boiling point (*T_bT I_* = 3287 °C), and *r*_0_ is the radius of the hole formed in the film. From (1):

(2) $$\Delta T_{0}=T_{mTi}\cdot \exp(2{r_{0}}^{2}/{\omega_{0}}^{2})-q_{1}/c_{1}-q_{2}/c_{2}$$

Using the tabular values of *q*_1_ = 15 kJ/mol, *q*_2_ = 425 kJ/mol, *c*_1_ = 37 J/(mol·K), and *c*_2_ = 46 J/(mol·K) [19] for *r*_0_ = 15 μm (Figure 3A) in Equation (2), we obtain Δ*T*_0_ ~ 2700 K. Because this value is lower than *T_bTi_* = 3287 °C, it can be assumed that at the center of the laser spot only a part of the Ti film was evaporated, where the maximum temperature reached *T_bTi_* = 3287 °C.

**Figure 4:**
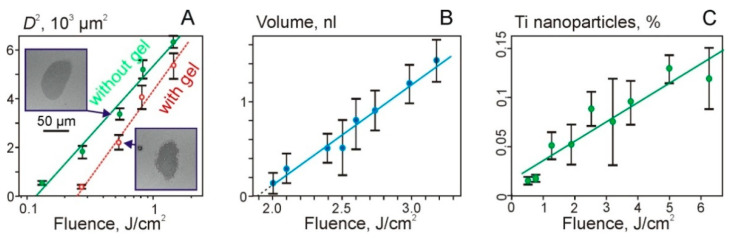
Dependences of the square of the hole diameter in Ti film (**A**), the volume of the transferred gel microdroplets (**B**), and the percentage of TiNP in microdroplets on the laser fluence (**C**). Figure 4A shows values for the Ti film with and without a gel layer (empty red circles). Examples of SEM images of holes for *F* = 540 mJ/cm^2^ are also shown.

Figure 4 shows some experimental results and dependences on the laser fluence. It can be seen that the dependences of the square of the hole diameter D in Ti film on the logarithm of the laser fluence are practically linear, both for the dry Ti film and for Ti film with a gel layer. This could be expected for Gaussian beams since it is easy to show that the following relation should be fulfilled:

(3) $$D^{2}=2\cdot {\omega_{0}}^{2}\cdot \ln(F_{0}/F_{th}),$$

where ω_0_ is the radius of the laser waist, *F*_0_ is the peak laser fluence at the center of the laser spot, and *F_th_* is the threshold fluence at which the destruction of the Ti film begins. From Equation (3), it follows that *D* = 0 at *F*_0_ = *F_th_*.

The intersection of linear dependences with the fluence axis in Figure 4A shows the values of the threshold fluence. Thus, we obtain that *F_th_* ~ 120 mJ/cm^2^ for the dry film and *F_th_* ~ 250 mJ/cm^2^ for the film with a gel layer. The approximately two-fold increase in the threshold for the destruction of the Ti film in the presence of gel on its surface can be explained by an additional heat transfer into the hydrogel layer.

The region of laser fluences shown in Figure 4B is described by a linear dependence: *V* = 1.1·*x* − 2, where *V* is the microdroplet volume in nanoliters and *x* is the laser fluence *F* in J/cm^2^.

Due to the destruction of the Ti film, TiNP can be transferred in microdroplets and deposited on the donor slide (Figure 4C). The dependence of the percentage of TiNP in a gel droplet on the laser fluence can be described as follows:

*P*1 = 0.019·*x* + 0.017 (4)

where *x* is the laser fluence *F* in J/cm^2^.

**Figure 5:**
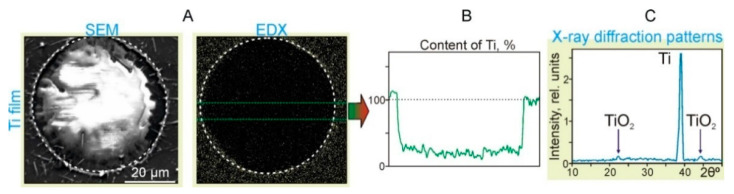
Results of the action of single laser pulses with the energy *E* = 19.5 mJ and laser fluence *F* = 2.8 J/cm^2^ on the Ti film located in close contact with the sapphire acceptor plate. (**A**) SEM image (SEM) and (**B**) the surface distribution map of titanium particles (EDX). The dotted circles denote an area with the diameter of 54 μm. (**C**) X-ray diffraction patterns of the Ti film in the region near the dotted circle.

In the second series of experiments, the action of single laser pulses on the donor slide with the Ti film and sapphire acceptor plates, which were placed in close contact, was studied. Figure 5A shows a SEM image of the surface of the donor slide after laser irradiation with a pulse energy of 19.5 mJ. A hole in the Ti film with the diameter of ~54 μm can be identified in this SEM image. Figure 5B also shows the corresponding maps of the distribution of titanium particles over the surface, obtained using EDX analysis.

It can clearly be seen in the Ti distribution map (EDX in Figure 5B) that the concentration of TiNP sharply decreases inside the region marked by the dotted circle. According to the graph, the content of Ti within the region is only 25% ± 5% of the value corresponding to the Ti film. At the same time, at the border of the region, the Ti content increased by 15% ± 5%. In the X-ray diffraction patterns of the Ti film near the hole edges (Figure 5C), in addition to the main Ti peak, there are two clearly distinguishable peaks associated with the formation of titanium oxides. Note that the mechanism of laser-induced oxidation of titanium during pulsed laser heating is well-described by the laser-induced Cabrera–Mott oxidation theory [19]. In this case, the rate of oxidation is determined by the migration rate of oxygen ions, which is largely influenced by the laser-induced Mott potential and temperature.

**Figure 6:**
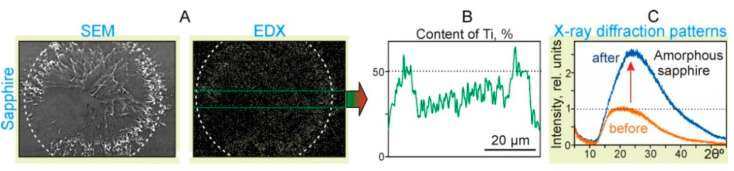
Results of the action of single laser pulses with the energy E = 19.5 mJ and laser fluence F = 2.8 J/cm^2^ on the sapphire acceptor plate placed in close contact with the Ti film on the donor slide. (**A**) SEM images (SEM) and surface distribution maps of Ti particles (EDX). The dotted circles denote an area with a diameter of 54 μm. (**B**) The surface distribution map of Ti particles. (**C**) X-ray diffraction patterns of the sapphire surface inside the dotted circle before and after the laser pulse exposure.

SEM image of the sapphire acceptor plate (Figure 6) also clearly shows a modified region of ~54 µm in diameter. An increased concentration of Ti particles in this region and near it edges is observed, which is confirmed by the EDX analysis data. According to the corresponding EDX graph (Figure 6B), the Ti content at the region boundary is significantly increased up to 58%. Inside this region, the Ti content has an average of 37% ± 12% compared to the Ti film. Thus, one can conclude that most of the Ti film material from the donor slide has been transferred onto the sapphire acceptor plate. Due to the high temperature and pressure at the hole center during laser ablation, the generated particles are scattered radially, forming an increased Ti concentration at the hole boundaries (Figure 5B and Figure 6B).

The X-ray diffraction patterns of the sapphire plate surface (Figure 6C) show that after the laser exposure, the content of the amorphous sapphire fraction increased significantly by ~2.5 times. It is well-known that the appearance of the amorphous phase of sapphire can happen due to short laser pulse heating, sapphire melting, and rapid cooling [22]. Due to the atomic structure difference between the crystal and liquid phases, during the cooling process phase, transformation consists of two major steps: nucleation (bulk nucleation) and crystal growth [23]. When the cooling rate is high, and there is no time for the nucleation process to be finished, polycrystallization or amorphization phases are produced. Thus, the obtained results prove that, in the region of laser pulse action, the temperature of the surface layer of sapphire exceeded its melting temperature *T_ms_* = 2040 °C.

**Figure 7:**
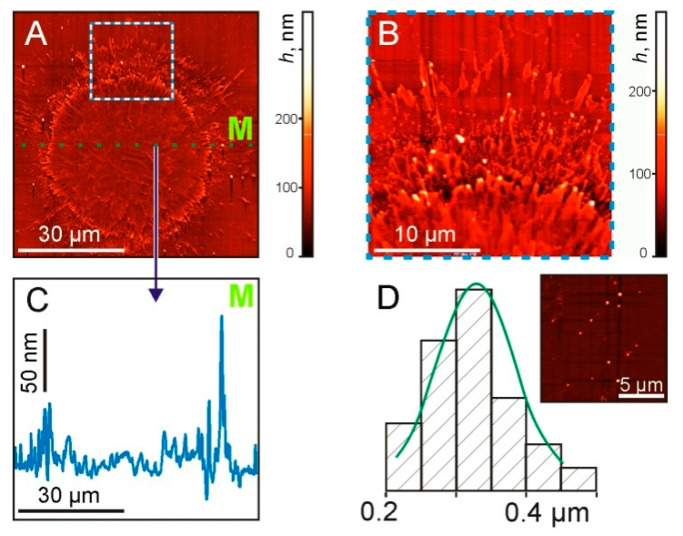
AFM images of the sapphire plate surface after the laser pulse exposure at the same laser parameters as in previous figures. (**A**) General view of the surface. (**B**) The enlarged part at the border area (marked by the square in (**A**)). (**C**) Cross-sectional profile along the dotted green line M in (**A**,**D**). The size distribution of TiNP. On the inset—a surface area with nanoparticles.

In Figure 7, AFM images of the sapphire acceptor plate with a clearly distinguishable increase in the Ti concentration in the region of ~50 μm diameter. At the edges of this region, submicron-scale structures with a width of 50–100 nm and directed from the central region are clearly visible.

The cross-sectional profile (Figure 7C), along the dotted green line M shown in Figure 7A, shows micron-size irregularities along the entire length. In this case, maximum peaks with a drop of up to 130 nm were recorded at the boundary of the irradiated region. The size distribution of TiNP with an average diameter of 330 ± 70 nm is shown in Figure 7D.

**Figure 8:**
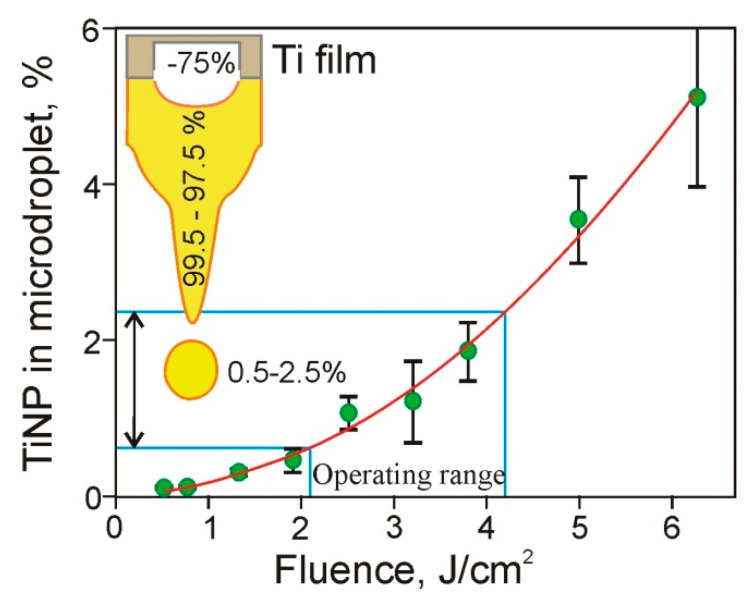
Dependence of the ratio (in percentages) of TiNP mass transferred in microdroplets to the mass of the ablated Ti film on the laser pulse fluence. The inset shows a titanium film, a jet of gel, and a microdroplet. The numbers on the Figure provide the percentage of material removed from the Ti film (~75%), as well as the percentage of the removed material located in the jet (99.5–97.5%) and in gel microdroplet (0.5–2.5%).

Using data obtained in the above experiments, we can define the ratio P2 (in percentages) of the total mass of TiNP transferred by the gel microdroplet to the mass of the ablated Ti film (corresponding to the hole). The obtained results are shown in Figure 8 and are well described by the simple quadratic equation:

*P*2 = 0.13·*x*^2^ + 0.03 (5)

where *x* is the laser pulse fluence *F* in J/cm^2^.

Using Equation (5), one can see that in the operating range of the laser pulse energies (15–30 μJ) used in the LEMS technology [17], only a small fraction (0.5% to 2.5%) of the Ti material ablated from the Ti film is transferred by the gel microdroplets. It is well-known that in laser printing, microdroplets detach from the tip of the gel jets, which are then returned to the donor slide [1,14,22]. Practically all nanoparticles formed due to the destruction of the Ti film remain in this jet and return with the jet in the hydrogel layer located on the donor slide surface. Only a small number of these TiNP are transferred in microdroplets to the acceptor slide. Thus, we can conclude that relatively thick layers of hydrogel [9,13,14] used in LEMS technologies protect living cells from shock waves [17] and, as it is shown in this paper, reduce the amount of metal nanoparticles in microdroplets.

In the presented work, we used a gel without microbiological objects, but we believe that the results obtained can be fully applied in laser printing with bioinks containing cells. The process of destruction of a metal film with the formation of nanoparticles is mainly determined by the material and parameters of this film, as well as by the parameters of laser action and the thermophysical characteristics of the gel layer. The addition to the hydrogel layer of a small concentration of cells or microorganisms will not lead to significant changes in its thermophysical characteristics. The obtained experimental results are important for the further development of laser bioprinting and LEMS technologies.

## 4. Conclusions

In this paper, investigations of the number of metal nanoparticles transferred with gel microdroplets during laser bioprinting have been performed. Metal nanoparticles transferred together with living cells and microorganisms can have significant effects on their metabolism and survival. Dependences of the hole size in the metal absorbing film, volumes of the formed microdroplets, and the number of TiNP within them on the laser pulse energy have been studied. It has been demonstrated that only an insignificant number of nanoparticles formed due to the destruction of the absorbing film is transferred with microdroplets to the acceptor slide (0.5% to 2.5%). Most of the nanoparticles remain in the gel layer on the donor slide. Using atomic force microscopy, the size distribution of the transferred nanoparticles has been defined. The obtained experimental results are important for the further development of laser bioprinting and LEMS technologies.
